# Prevalence of motorcycle accidents and associated factors among road traffic accident victims in Accra, Ghana

**DOI:** 10.4314/gmj.v59i1.1

**Published:** 2025-03

**Authors:** Victoria A F Luther, Delia AB Bandoh, Adolphina A Addo-Lartey

**Affiliations:** 1 Department of Epidemiology and Disease Control, University of Ghana, School of Public Health. Legon, Accra, Ghana

**Keywords:** Motorcycle, accidents, risk factors, prevalence, Ghana

## Abstract

**Objectives:**

This study determined the prevalence of motorcycle accidents and associated risk factors in Accra, Ghana.

**Design:**

Cross-sectional

**Setting:**

Legon, Pentecost, Madina, and Kekele hospitals, Motor Transport and Traffic Unit offices in Accra, and Kaneshie District Court. Data were collected using structured questionnaires

**Participants:**

Road traffic accident victims (387), aged 15 years and above, were randomly selected from health facilities' registers and interviewed.

**Main outcome measure:**

Number of road traffic accidents involving motorcycles

**Results:**

The prevalence of motorcycle accidents among road traffic accidents was 37% [95% CI: 32.0-42.0]. Motorcycle accidents predominantly occurred among people aged 15-25 and 26-35 years. The prevalence among men was 38% while women reported 24% [95% CI: 85.0-91.0]. Motorcycle accident occurrence was higher (63% vs 10%) among those earning < GH₵349 ($22.37) a month compared to those earning above GH₵3000 ($192.31). After adjusting for all the factors that showed association at the univariate level and potential confounders like sex, followed by post-estimation analysis and a Hosmer-Lemeshow goodness of fit test, factors that were significantly associated with motorcycle accidents included ownership [AOR:2.32, p=0.018 95%CI: 1.16-4.65], education level [AOR=1.48, p=0.020 95%CI: 1.06-2.05], motor license [AOR=132.74, p<0.001 95%CI: 17.56-1003.62], and income level [AOR=0.65, p=0.002 95%CI: 0.50-0.85].

**Conclusion:**

Motorcycle accidents remain prevalent and significantly related to income level, ownership level, education level, and having a motor license. To reduce motorcycle accidents, governments must implement policies that address these factors and support safer road practices.

**Funding:**

None declared

## Introduction

Road Traffic Accidents (RTAs) are an emerging Public Health issue and the eighth cause of mortality in all age categories.^1^, ^2^ It is the leading cause of death among those aged 5 to 29 worldwide 1, 2, 3 Motorcycle accidents account for more than 380,000 deaths per year globally, accounting for 28% of all road fatalities.^1^ Developing countries bear the greatest brunt. Low- and middle-income countries account for over 90% of road traffic fatalities.^1^,^4^ The prevalence of motorcycle accidents among all traffic accidents in East Africa is 21.0%–58.8%. 5 Previous reported prevalence of motorcycle accidents ranges from 22.8% in China^6^ to 62% in Vietnam^6^, 50.4% to 55.1% in Ethiopia^7^, ^8^ and 61.5% in Kenya.^9^

RTA deaths in Ghana reached 7,472 in 2018, accounting for 3.73% of overall deaths.^1^ According to data compiled by the Ghana Police Service's Motor Traffic and Transport Department (MTTD), motorcycle accidents increased by 12.74% from 3,487 in 2017 to 3,903 in 2018.^10^ Accra, Ghana's capital city, reported most of these cases, with 5,514 cases accounting for approximately 42.0% of total cases. 10

In Ghana, there is increasing utilisation of motorcycles for transportation.^11^ This high utilization can be attributed to its ease of navigation or swiftness in traffic congestion and affordability for those with less income and convenience.^12^

Motorcycle accidents have social, physical, and economic consequences for countries, families, and individuals.^13^ Motorcycle accidents affect economically active people and incur financial expenditures for the nation and the afflicted. RTAs result in avoidable premature deaths, injuries, disabilities, and hospitalization of the victims. RTAs also have a social impact on the affected individual's relatives and friends because they may have to bear or support the financial costs for the victim. Additionally, the death of a loved one in a motorcycle accident could be terrible and difficult for family and friends, especially if the loved one was a breadwinner.

The National Road Safety Commission (NRSC) and the Motto Traffic and Transport Department of the Ghana Police Service have been conducting road safety campaigns and enforcing road safety laws to help reduce this problem in Ghana. However, more can be done, especially in research by individuals and organizations, to help inform policy.

Despite being a serious public health concern, few studies have been conducted in this field, notably in Accra, Ghana's capital city, where the burden is high: 5,514 representing approximately 42.0% of RTAs.^10^ The few studies conducted in Ghana included municipalities such as Bolgatanga and Techiman. The societal cost of motorcycle accidents was studied in the municipality of Bolgatanga, where motorbike accidents cost the municipality GH 1,630,979.60 (US $ 104,549.97) per year in terms of social costs. This was in addition to the pain, sorrow, suffering, and in severe cases lifelong impairment.^6^ Another study conducted in the Techiman Municipality focused on identifying risk factors in Ghanaian road traffic accidents.^14^ According to Awal's research, many RTA fatalities are the result of avoidable human errors.^14^

This research sought to provide new information to inform policy development and enforcement to prevent motorcycle accidents. The objective of this study was to investigate the prevalence of motorcycle accidents and the factors that contribute to them among victims of road traffic accidents in Accra, Ghana.

## Methods

### Study design

A cross-sectional study among motorcycle accident victims aged fifteen years and above who had been involved in accidents from December 2018 to May 2019 (6 months) was carried out in Accra. A structured questionnaire was used to collect data through face-to-face interviews for 6 weeks from May 2019 to June 2019). All accident victims from The Ghana Police Service's Motto Transport and Traffic Department, Pentecost Hospital, Legon Hospital, Madina Polyclinic, Kekele Hospital, and Kaneshie District Court were eligible to participate. Persons engaged in motorcycle accidents were identified and recruited from the RTA victims at the selected health facilities and institutions. These locations were chosen because they are in the country's capital city, Accra, which is densely inhabited by people from all over the country. Individuals involved in accidents were also more likely to report to a health facility, a police station, or a courthouse.

### Study location

Accra is the capital city of Ghana. It is Ghana's most densely populated metropolis, with a population of 2,052,341 people.^15^ Accra serves as the economic and administrative centre for the Greater Accra Region and the capital city of Ghana. The main modes of transportation in Accra are ‘trotro’ (minivans), buses, taxis, and ‘okada’ (motorcycles).

Accra was chosen for this study because of its cosmopolitan nature, with residents from all around the country as well as foreigners from an array of countries. Most importantly, traffic congestion in Accra can be extreme, causing vehicles and passengers to be stuck on the road for hours, merely to go a short distance. Due to the heavy traffic situation, motorcycles have been used as a mode of transportation to avoid traffic congestion.

### Study population

Victims of motorcycle accidents (riders, passengers, drivers and pedestrians) above 15 years of age from December 2018 – May 2019, that reported at Legon Hospital, Madina Polyclinic, Kekele Hospital, or Pentecost Hospital, or victims that reported at MTTU and Kaneshie district court and were capable of narrating their experiences were enrolled.

### Sample size estimation

The sample size was determined using the Cochran formula.^16^ Motorcycles are responsible for 32% of total traffic accidents, according to a study in Singapore^17^ As a result, a sample size of 354 was determined using a prevalence of 36% and a margin of error of 5% at a 95% confidence interval. A 5% non-response rate was added to account for anticipated non-response and incomplete surveys, yielding a minimum sample size of 372.

### Sampling of participants

A list of all persons involved in a road traffic collision or accident within the period of December 2018 to May 2019 was obtained from Pentecost Hospital, Legon Hospital, Madina Polyclinic, Kekele Hospital, MTTU, and Kaneshie District Court. This comprised passengers, riders, drivers, and pedestrians injured in traffic accidents. The lists from the various sites were compiled as a single list and numbered. Using the random number generation command in Microsoft Excel, 372 numbers from the compiled list of accident victims were randomly generated, and the corresponding victims were selected for interview. The selected victims were contacted and recruited into the study upon agreeing to take part. All those who declined were replaced.

### Inclusion criteria

The inclusion criteria were being a victim of a traffic accident, a driver, rider, passenger or a pedestrian aged 15 years and above who reported to any of the study sites and was admitted or discharged between December 2018 and May 2019.

### Exclusion criteria

The study excluded eligible motorcycle accident victims who were in critical condition or died during the accident.

### Data collection

Selected victims who agreed to take part in the study were contacted, and interview appointments were booked. On the day of the interview, informed consent (for adults) and assent of the accident victims (for those under 18 years) were obtained from each participant. Study participants were then interviewed face-to-face by trained research assistants. A structured, interviewer-administered questionnaire was used for the collection of data. Data collected included information on participants' demographics, riding history, type of accident, and factors that could influence motorcycle accidents such as income level, level of education, and maintenance frequency. The questionnaire was carefully developed by researchers based on researched relevant literature on motorcycle accidents and sociodemographic factors that could influence motorcycle accidents.^5^,^8^,^14^ The questionnaire was validated by conducting a pilot test on 20 victims from another health facility within the Greater Accra region; checking for reliability, surface, content, criteria and construct validity. Based on that, the questionnaire was revised before the main study was conducted. The pilot study was conducted from 13/05/2019 to 15/05/2019. Interviews were done on the premises of the institutions. The questions were explained in the Twi or Ga language for those who did not grasp the questions or the English language. To ensure that translating the questionnaire to Twi or Ga did not change the interpretation in English, the questionnaires were first translated into the two local languages. A back translation was done for both languages to ensure the integrity of the question was maintained during the translation. Research assistants who were fluent in these languages were then trained by an expert committee in the translated versions of the questionnaire before the start of the study. The data was collected over six weeks (from May 2019 to June 2019), after which it was analysed.

### Data analysis

Data were initially entered into Microsoft Excel (2013) before being imported and analysed in Stata (version 15). For categorical variables, frequencies and percentages were calculated. For continuous variables, means and standard deviations were computed. Chi-square tests were used to test the relationships between motorcycle accidents and demographic or socioeconomic characteristics. Bivariate and multivariable logistic regression analyses were performed to assess the association and strength of association between outcome and exposure variables. The multivariate logistic regression adjusted for potential confounders of the above associations and included all variables significantly associated with motorcycle accidents in the bivariate analyses. Potential confounding factors such as age and sex were accounted for in the analysis by controlling for them. This was followed by post-estimation analysis to determine the overall effect of variables on motorcycle accidents. A Hosmer-Lemeshow goodness of fit test was also conducted to determine whether the fitted model adequately described the observed outcome experience in the data. All p-values less than 0.05 were deemed statistically significant.

### Ethical considerations

The Ghana Health Service Ethical Review Committee approved the study (GHSERC: 040/02/19). The La-Nkwantanang Madina Municipal Assembly, the MTTU, Kaneshie District Court, and the Greater Accra Regional Health Directorate granted permission to conduct the research at the selected facilities. Participants aged 18 years and above provided informed consent. Parents or guardians of participants aged 15 to 17 years provided informed assent.

## Results

### Characteristics of the study participants

The study included 387 victims of road traffic accidents. Among them, 341 (88%) were males. The male-to-female ratio was 341(88%) males and 46 (12%) females. The majority of these victims of road traffic accidents interviewed 156 (40%) were between the ages of 26 and 35. The average age was 35 years, with a standard deviation of 9.98. The majority of participants 158 (41%) had a secondary/vocational education, and 198 (51%) were self-employed (Table 1).

**Table 1:** Characteristics of victims of road traffic accidents in Accra, Ghana from December 2018 - May 2019, (N=387)

Characteristics	Frequency, n (%)
**Age**	
15-25	49 (12.7)
26-35	156 (40.3)
36-45	127 (32.8)
46-55	47 (12.1)
56-65	8 (2.1)
**Sex**	
Female	46 (11.9)
Male	341 (88.1)
**Education level**	
Tertiary	112 (28.9)
secondary/vocational	158 (40.8)
middle/JHS	75 (19.4)
primary/elementary	27 (7.0)
None	15 ((3.9)
**Employment status**	
formal employment	79 (20.4)
self-employed	198 (51.2)
wage/seasonal employment	86 (22.2)
unemployed	24 (6.2)
**Income level GH₵ (US $)**	
0-349 (0-22.37)	57 (14.7)
350-649 (22.44-41.60)	138 (35.7)
650-949 (41.67-60.83)	56 (14.5)
950-1299 (60.90-83.27)	88 (22.7)
above 3000 (192.31)	48 (12.4)
**Family size**	
1 to 2	82 (21.2)
3 to 4	176 (45.5)
5 to 6	97 (25.1)
7 to 8	16(4.13)
9 to 12	16 (4.13)

### Prevalence of motorcycle accidents

Out of the 387 participants, 142 (37% [95% CI=32.0-42.0]) were involved in motorcycle accidents, hence a prevalence of 37% for motorcycle accidents among road traffic accident victims.

### Association of motorcycle accidents with sociodemographic and socioeconomic factors

A chi-square test of association at a 95% confidence interval and significance at p<0.05 was conducted to determine possible associations of sociodemographic and socioeconomic factors with the outcome (motorcycle accidents). From Table 2, males were more likely to be involved in a motorcycle collision (39%, n=132/341) than females (22%, n=10/46). Motorcycle accidents occurred at a frequency of 61% (30/49) among persons aged 15 to 25. The frequency was 80% (12/15) among people with no formal education. Persons with a monthly income of 0 - 349 Ghana cedis had a high motorcycle accident prevalence of 63% (36/57). The chi-square test of association indicated that age (p<0.001), income (p<0.001), family size (p<0.001), license (p<0.001), frequency of maintenance (p<0.001), insurance (p<0.001), education level (p=0.001), and employment (p<0.001) were all significantly associated with motorcycle accidents (Table 2).

**Table 2:** Associations between socio-demographic and motorcycle accidents in Accra

Variables	Motorcycle accidents (%)	Car accidents (%)	χ^2^	p-value
**Age**			21.11	< 0.001
15-25	30 (61.2)	19 (38.8)		
26-35	62 (39.7)	94 (60.3)		
36-45	38 (29.9)	89 (70.1)		
46-55	10 (21.3)	37 (78.7)		
56-65	2 (25)	6 (75)		
**Sex**			3.67	0.055
Female	11 (23.9)	35 (76.1)		
Male	131 (38.4)	210 (61.6)		
**Educational level**			50.3	<0.001
tertiary	13 (11.6)	99 (88.4)		
secondary/vocational	70 (44.3)	88 (55.7)		
middle/JHS	35 (46.7)	40 (53.3)		
primary/elementary	12 (44.4)	15 (55.6)		
None	12 (80.0)	3 (20.0)		
**Employment status**			27.56	< 0.001
formal employment	13 (16.5)	66 (83.5)		
self-employed	94 (47.5)	104 (52.5)		
wage/seasonal	24 (27.9)	62 (72.1)		
unemployed	11 (45.8)	13 (54.2)		
**Income level, GH₵ (US $)**			58.84	< 0.001
0-349 (0-22.37)	36 (63.2)	21 (36.8)		
350-649 (22-42)	69 (50.0)	69 (50.0)		
650-949 (42-61)	18 (32.1)	38 (67.9)		
950-1299 (61-83)	14 (15.9)	74 (84.1)		
above 3000 (192)	5 (10.4)	43 (89.6)		
**Family size**			22.49	< 0.001
1 to 2	37 (45.1)	45 (54.9)		
3 to 4	62 (35.2)	114 (64.8)		
5 to 6	25 (25.8)	72 (74.2)		
7 to 8	5 (31.3)	11 (68.8)		
9 to 12	13 (81.2)	3 (18.8)		
**Owning of motorcycle/car**			2.87	0.090
no	38 (30.7)	86 (69.4)		
yes	104 (39.5)	159 (60.5)		
**Insurance**			4.78	0.029
no	27 (50.0)	27 (50.0)		
yes	115 (34.5)	218 (65.5)		
**Maintenance frequency**			334.42	< 0.001
never	0 (0.0)	10 (100.0)		
monthly	0 (0.0)	107 (100.0)		
every three months	1 (1.3)	74 (98.7)		
every six months	1 (3.5)	28 (96.6)		
annually	0 (0.0)	2 (100.0)		
when car/bike breaks down	1 (6.7)	14 (93.3)		
**License to drive/ride**			125.85	< 0.001
no	79 (24.5)	244 (75.5)		
yes	63 (98.4)	1 (1.6)		
				

### Strength of association of motorcycle accidents with sociodemographic/socioeconomic factors

Bivariate logistic regression analysis was also performed to show factors associated with motorcycle accidents, after which the strength of association was determined using multivariate logistic regression analysis. As shown in Table 3, bivariate analysis showed that age, maintenance frequency, license for motor, education level, income level and insurance were related to motorcycle accidents significantly(p<0/05). The crude odds of motorcycle accidents among those with income levels above 3000 were 0.07 times the odds of motorcycle accidents among those with income levels 0 to 349 (COR=0.07, p<0.001 95%CI 0.02-0.20). The odds of motorcycle accidents among those whose family size consists of 9 to 12 members was 5.27 times the odds of motorcycle accidents among participants with family size 1-2 members. (COR=5.27, p=0.012 95%CI 1.40-19.90) Among participants who had insurance, the odds of motorcycle accidents were 0.53 times the odds of motorcycle accidents among participants who did not have insurance. (COR=0.53, p=0.030 95%CI 0.30-0.94)

**Table 3:** Strength of association of risk factors with motorcycle accidents in Accra

Variables	Crude odds ratio (COR)	95% CI	Adjusted odds ratio (AOR)	95% Confidence interval (95% CI)
**Sex**	1.98	0.97-4.04	0.69	0.27-1.79
Female	1			
Male	6.17	0.97-4.04	0.50	0.15-1.63
**Age group in years**	0.60	0.47-0.76**	0.72	0.51-1.03
15-25	1			
26-35	0.42	0.22-0.81*	0.43	0.16- 1.15
36-45	0.27	0.14-0.54**	0.34	0.11 - 1.03
46-55	0.17	0.07-0.42**	0.29	0.07 - 1.23
56-65	0.21	0.04-1.16	1.89	0.22 - 16.55
**Family size**	1.09	0.90-1.31	0.98	0.70- 1.36
1 to 2	1		1	
3 to 4	0.66	0.39-1.13	0.89	0.40-1.96
5 to 6	0.42	0.23-0.79*	0.77	0.29-2.05
7 to 8	0.55	0.18-1.73	0.53	0.10-2.89
				
9 to 12	5.27	1.40-19.90*	3.56	0.27-47.32
**Highest educational level**	1.88	1.51-2.34**	1.48	1.06-2.05*
tertiary	1		1	
secondary/vocational	6.06	3.14 - 11.70**	5.23	1.86 -14.68*
middle/JHS	6.66	3.20 - 13.90**	5.24	1.67 -16.45*
primary/elementary	6.09	2.35 -15.82**	2.70	0.53 -13.78
none	30.46	7.58 -122.42**	14.05	1.25 -158.45*
**Employment status**	1.24	0.96-1.60	0.93	0.64-1.35
formal employment	1		1	
self-employed	4.59	2.38-8.85**	1.30	0.50-3.41
wage/seasonal employment	1.97	0.92 -4.20	1.03	0.33-3.18
unemployed	4.30	1.58-11.67*	1.07	0.23-4.92
**Income level, GH₵(US$)**	0.48	0.39-0.59**	0.65	0.50-0.85*
0-349 (0-22.37)	1		1	
350-649 (22.44-41.60)	0.58	0.31-1.10	0.71	0.30-1.70
650-949 (41.67-60.83)	0.28	0.13-0.60*	0.26	0.08-0.81*
950-1299 (60.90-83.27)	0.11	0.05-0.24**	0.22	0.07-0.68*
above 3000 (192.31)	0.07	0.02-0.20**	0.23	0.05 -1.08
**Ownership**	1.48	0.94-2.33	2.32	1.16-4.65*
No	1		1	
Yes	1.48	0.94-2.33	2.95	1.33-6.55**
**Insurance**	0.53	0.30-0.94*	0.48	0.19-1.21
No	1			
Yes	0.53	0.30-0.94*	1.00	0.31-3.30
**Maintenance frequency**	1.47	1.28-1.71**	1.41	1.15-1.72*
Never	1		1	
Monthly	0.51	0.24-1.08	0.72	0.18-2.87
Every three months	0.28	0.12-0.65*	0.28	0.06-1.18
Every six months	0.89	0.37-2.14	0.45	0.10-2.02
Annually	3.75	0.66-21.15	1.91	0.18-19.89
When car/bike breaks down	4.06	1.62-10.22*	3.61	0.81-16.10
**License**	194.58	26.55-1425.87**	132.74	17.56-1003.62**
No	1		1	
Yes	194.58	26.55-1425.87**	184.03	19.21- 1762.73**

* p-value <0.05

** p-value <0.001

#variables adjusted for in the model were age, education level, employment status, family size, insurance, motor licence, ownership, maintenance frequency, income level and sex.

After adjusting for all the variables that were significant in the bivariate model and potential confounding factors like sex, using a multivariable logistic regression model, only ownership [AOR:2.32, p=0.018 95%CI: 1.16-4.65], education level [AOR=1.48, p=0.020 95%CI: 1.06-2.05], motor license [AOR=132.74, p<0.001 95%CI: 17.56-1003.62], and income level [AOR=0.65, p=0.002 95%CI: 0.50-0.85] remained significantly linked with motorcycle accidents (p<0.05). After post-estimation analysis, employment status did not show an overall significant effect on motorcycle accidents (p>0.05). Table 3 demonstrates these findings

### The Hosmer-Lemeshow goodness of fit test

A Hosmer-Lemeshow goodness of fit test was conducted and yielded a coefficient of 269.28, a p-value of 0.954 indicating that the fitted model adequately describes the observed outcome experience in the data.

## Discussion

Motorcycle accidents have grave public health and economic ramifications in developing countries, including Ghana. This study looked at the frequency of motorcycle accidents and the risk factors that contribute to them. According to the study's findings, males made up the vast majority of vehicle accident casualties. This is consistent with studies conducted in Tanzania, Cameroon, Malaysia, and Iran, which showed that more males were involved in motorcycle accidents than females.^18^-^21^ Males, as the primary breadwinners in most African families, are disproportionately affected by RTA, which can have a negative impact on their role and, as a result, their dependents when such incidents occur. 22-24 Males are the most affected, which can be explained by their risk-taking behaviour compared to females, who generally tend to be more cautious. 18-20, 24, 25

This implies that policymakers will have to target males more with effective policies to help reduce motorcycle accidents.

Persons who had the most motorcycle accidents were between the ages of 15 to 25 and 26 to 35. This observation is consistent with the age ranges suggested in other studies.^1^, ^20^, ^26^-^28^ This could be related to the youth's proclivity to be more adventurous and to take greater risks, such as unsafe driving.^29^, ^30^ This means that the youth are more likely to be involved in motorbike accidents. As a result, extreme efforts must be made by the government and civic communities to combat this threat by educating the youth on the dangers of motorcycle accidents and the need for prevention

This study found that motorcycle accidents accounted for more than one-third of all road accidents. The study findings are comparable to reports in Kenya, Tanzania, Ethiopia, China, and Vietnam that found that Motorcycle accident prevalence ranges from 22.8% to 62%.^6^-^9^ This can be explained by the increased usage of motorbikes as a preferred mode of transit to avoid traffic congestion and as a method of commercial employment.^31^-^36^

This implies that with the upsurge of motorcycles, more accidents may occur if nothing is done. Hence policies should be put in place by the government to ensure that only those with proof that they can ride safely should be allowed to ride motorcycles. Motorcycle accidents were nearly twice as common among males as they were among females (males= 39%: females=22%). This contrasts with a study conducted by Cavalcanti et al., 21 among victims of motorcycle accidents in a trauma centre in Brazil, which found that men were two times more likely than women to be involved in motorcycle accidents. Males are likely more affected than females because of their risk-taking behaviour of driving faster and more carelessly. 7, 29, 30, 37 Hence education programs by civic groups on motorcycle accidents should be targeted mainly at males to help reduce motorcycle accidents.

The results demonstrate a significant association between motorcycle accidents and a wide range of sociodemographic/socioeconomic characteristics at the univariate level, which is comparable to other studies in Africa and Europe.^7^, ^9^, ^38^-^40^ These studies showed that people from lower socioeconomic status groups have a higher risk of being involved in road crashes thus socioeconomic and sociodemographic factors were associated with motorcycle accidents. The reason for this similarity could be that persons in low socioeconomic brackets may not regard or border the risk of motorcycle accidents, basically because they are affected by other existing more serious social issues.^41^ This implies that socioeconomic status can affect motorcycle accidents. Hence, to address motorcycle accidents, policymakers must tackle people with low socioeconomic levels.

However, after adjusting for all potential confounders like sex, only income level, ownership, education level and motor licence were still significantly associated with motorcycle accidents (p<0.05). This is because they are independent predictors. The remaining variables (age, sex, insurance, employment status, maintenance, and family size) were no longer significantly associated with motorcycle accidents. This is comparable with many studies in Africa, Asia and Europe, that show that income level and educational level are associated with motorcycle accidents. 42, 43-45 The reduced odds of motorcycle accidents among those with higher income, compared to those with less income could be attributed to motorcycle transportation being cheaper and more affordable^32^ for those with lower income levels. Additionally, those with lower incomes may not be able to afford regular and adequate maintenance of motorcycles. Ownership being associated with motorcycle accidents is similar to a study in Tanzania among commercial motorcyclists that showed that not owning one's motorcycle was associated with higher risks of motorcycle injuries.^46^

All these mean that in addressing the issue of motorcycle accidents, the government and stakeholders will have to tackle education, acquisition of motor licenses, motorcycle ownership and the income level of the people concerned.

According to the study, motorcycle accidents are prevalent, especially among men and the youth (26-35 years) and are significantly related to income level, unemployment, ownership and maintenance frequency of motorcycles. The findings from this research imply that, if nothing is done to curb these motorcycle accidents, those who are not rich (financially), most breadwinners of families (mainly men), and the economically active workforce (who form most of the population), may continue to be adversely plagued with motorcycle accidents and ultimately may lead to their death. Findings from the research can be utilized by policymakers to make and enforce policies that address the issues of education, income level, licence acquisition and maintenance frequency to help curb motorcycle accidents.

One limitation of the study was the possibility of recall bias since participants had to recall incidents in the past. However, steps were taken to reduce it to an extent by including only participants who had been involved in a road accident no more than six months ago. Another limitation is that with a cross-sectional study, the timing of collecting the data is not guaranteed to indicate temporal associations; hence, causality cannot be inferred.

This study is useful for establishing preliminary evidence for planning a future advanced study. It also allows one to compare many different variables at the same time. Since a representative sample was used for the research, the study findings can be used to make generalisations about the population.

It is recommended that motorists be educated by the MTTU and other relevant stakeholders on the need to adhere to road safety regulations such as driving with a valid license, maintaining their cycles and obeying road safety laws during riding. The Ministry of Health should help address this issue by using public health sensitization programs on motorcycle accidents that are targeted at the youth, males, the less educated and persons with low-income levels since they are more affected. Also, the government should reduce exposure by encouraging motorcycle riders and users to use safer modes of transport. Future research may consider using more rigorous designs to ascertain causal relationships between exposure variables and motorcycle accidents.

## Conclusion

The prevalence of motorcycle accidents in Accra was high with most victims being males. Motorcycle accidents predominantly affected the youth, the non-educated, and those with low-income levels. Socioeconomic and sociodemographic factors such as income level, education level, having a motor license and ownership were significantly associated with motorcycle accidents.

## References

[1] World Health Organization (2018). Global status report on road safety.

[2] Ramya M, Jadhav J, Ranganath T (2017). A study to determine the awareness and behavioural patterns/practice about road safety measures among undergraduate medical students, Bangalore, India-cross sectional study. Int J Community Med Public Health (Gujarat).

[3] World Health Organization (2015). Global status report on road safety.

[4] Schlottmann F, Tyson AF, Cairns BA, Varela C, Charles AG (2017). Road traffic collisions in Malawi: Trends and patterns of mortality on scene. Malawi Med J.

[5] Raga A, Asres AW, Samuel S, Addisu T, Abreha S (2023). Motorcycle accident and associated factors among commercial motorcycle drivers in Kindo Koyisha Woreda, Southern Ethiopia. The Open Transportation Journal.

[6] Odiwuor, Cholo Wilberforce, Nyamusi Esther, Odero Wilson (2015). Incidence of road traffic crashes and pattern of injuries among commercial motorcyclists in Naivasha Town. IJAR.

[7] Tadesse F, Mekonnen S, Kediro G, Gobena N, Bedaso A, Ebrahim J (2019). Prevalence and associated factors of motorcycle accident injuries in public Hospitals of Southern Ethiopia.

[8] Oltaye Z, Geja E, Tadele A (2021). Prevalence of motorcycle accidents and its associated factors among road traffic accident patients in Hawassa University Comprehensive Specialized Hospital, 2019. Open Access Emerg Med.

[9] Matheka DM, Omar FA, Kipsaina C, Witte J (2015). Road traffic injuries in Kenya: a survey of commercial motorcycle drivers. Pan Afr Med J.

[10] MTTD (2018). Ghana Police Service, Motor Traffic and Transport Department (MTTD) of the Ghana Police. Annual Report.

[11] Alimo PK, Rahim AB, Lartey-Young G, Ehebrecht D, Wang L, Ma W (2022). Investigating the increasing demand and formal regulation of motorcycle taxis in Ghana. Journal of Transport Geography.

[12] Kigotho JN (2021). Distribution, Patterns and Severity of Musculoskeletal Injuries Among Motorcycle Crash Victims at Kenyatta National Hospital. Unpublished Thesis.

[13] Sapkota D, Bista B, Adhikari SR (2016). Economic costs associated with motorbike accidents in Kathmandu, Nepal. Front Public Health.

[14] Mohammed A, Haadi A-R, Deen AJ (2015). Identification of risk factors involved in road accidents in Ghana. A case study of the Techiman municipality. Int J Stat Appl 2015.

[15] Ghana Statistical Service (2019). Demography: population projection.

[16] Cochran WG (1977). Sampling techniques.

[17] Haque MM, Chin HC, Huang H (2009). Modeling fault among motorcyclists involved in crashes. Accident Analysis and Prevention.

[18] Boniface R, Museru L, Kiloloma O, Munthali V (2016). Factors associated with road traffic injuries in Tanzania. Pan Afr Med J.

[19] Agbor AM, Chinedu AC, Ebot-Tabi B, Naidoo S (2014). Dentofacial injuries in commercial motorcycle accidents in Cameroon: pattern and cost implication of care. Afr Health Sci.

[20] Konlan KD, Hayford L (2022). Factors associated with motorcycle-related road traffic crashes in Africa, a Scoping review from 2016 to 2022. BMC Public Health.

[21] Cavalcanti AL, Lucena B, Rodrigues IS, Silva AL, Lima TT, Xavier AFC (2013). Motorcycle accidents: morbidity and associated factors in a city of the northeast of Brazil. Tanzan J Health Res.

[22] Akanle O, Nwaobiala UR (2020). Changing but fragile: Female breadwinning and family stability in Nigeria. J Asian Afr Stud.

[23] Setrana MB, Kleist N (2022). Gendered dynamics in West African migration. Migration in West Africa: IMISCOE Regional Reader.

[24] Abegaz T, Gebremedhin S (2019). Magnitude of road traffic accident-related injuries and fatalities in Ethiopia. PLoS One.

[25] Meyers-Levy J, Loken B (2015). Revisiting gender differences: What we know and what lies ahead. J Consum Psychol.

[26] Ospina-Mateus H, Quintana Jiménez LA, Lopez-Valdes FJ (2020). Understanding motorcyclist-related accidents in Colombia. Int J Inj Contr Saf Promot.

[27] Delamou A, Kourouma K, Camara BS, Kolie D, Grovogui FM, El Ayadi AM (2020). Motorcycle accidents and their outcomes amongst victims admitted to health facilities in Guinea: a cross-sectional study. Adv Prev Med.

[28] Ingabire JA, Petroze R, Calland F, Okiria J, Byiringiro J (2015). Profile and economic impact of motorcycle injuries treated at a university referral hospital in Kigali, Rwanda. Rwanda Med J.

[29] Benotsch EG, Snipes DJ, Martin AM, Bull SS (2013). Sexting, substance use, and sexual risk behaviour in young adults. J Adolesc Health.

[30] Mahalik JR, Levine Coley R, McPherran Lombardi C, Doyle Lynch A, Markowitz AJ, Jaffee SR (2013). Changes in health risk behaviours for males and females from early adolescence through early adulthood. Health Psychol.

[31] Konlan KD, Doat AR, Mohammed I, Amoah RM, Saah JA, Konlan KD (2020). Prevalence and pattern of road traffic accidents among commercial motorcyclists in the Central Tongu District, Ghana. Sci World J.

[32] Agyemang W, Adanu EK, Jones S (2021). Understanding the factors that are associated with motorcycle crash severity in rural and urban areas of Ghana. J Adv Transp.

[33] Salum JH, Kitali AE, Bwire H, Sando T, Alluri P (2019). Severity of motorcycle crashes in Dar es Salaam, Tanzania. Traffic injury prevention.

[34] Dapilah F, Guba BY, Owusu-Sekyere E (2017). Motorcyclist characteristics and traffic behaviour in urban Northern Ghana: Implications for road traffic accidents. J Transp Health.

[35] Fevriera S, de Groot HL, Mulder P (2021). Does Urban Form Affect Motorcycle Use? Evidence from Yogyakarta, Indonesia. Bulletin of Indonesian Economic Studies.

[36] Khan-Mohammad G (2023). Chinese Goods and mass consumption in Africa: Cultural appropriation of Chinese motorcycles in Burkina Faso. Critical African Studies.

[37] Erenler AK, Gümüş B (2019). Analysis of road traffic accidents in Turkey between 2013 and 2017. Medicina.

[38] Berghe VDW (2017). The association between road safety and socioeconomic situation (SES). An international literature review.

[39] Adeleye AO, Clark DJ, Malomo TA (2019). Trauma demography and clinical epidemiology of motorcycle crash-related head injury in a neurosurgery practice in an African developing country. Traffic Inj Prev.

[40] Camilloni L, Farchi S, Chini F, Rossi GP, Borgia P, Guasticchi G (2013). How socioeconomic status influences road traffic injuries and home injuries in Rome. Int J Inj Contr Saf Promot.

[41] Ajala O, Olabamiji A, Kalu C (2020). Livelihood and hazard vulnerability of commercial motorcycle and tricycle riders in Orile-Iganmu area, Lagos state, Nigeria. Ethiopian J Environ Stud & Manag.

[42] Munene BK (2022). Motorcycle Crashes and Socio-economic and Demographic Characteristics of Riders. A Case Study of South Imenti Sub County, Meru County. Unpublished Thesis.

[43] Sahr Nyuma E, Lawrence Sao B JS (2020). The Factors Associated with the Occurrence of Road Traffic Accidents among Commercial Motorcycle Riders in Kenema City, Eastern Sierra Leone. J Public Health Dis Prev.

[44] Almeida GCMd, Medeiros FdCDd, Pinto LO, Moura JMBdO, Lima KC (2016). Prevalence and factors associated with traffic accidents involving motorcycle taxis. Revista brasileira de enfermagem.

[45] Shaker R, Eldesouky RS, Hasan O, Bayomy H (2014). Motorcycle crashes attitudes of the motorcyclists regarding riders' experience and safety measures. J Community Health.

[46] Francis F, Möller J, Moshiro C, Kiwango G, Yngve BH, Hasselberg M (2023). Associations and mediators between types of motorcycle ownership and road traffic injuries among motorcycle taxi drivers in Dar es Salaam, Tanzania. Safety science.

